# Detection and Discrimination of Bacterial Colonies with Mueller Matrix Imaging

**DOI:** 10.1038/s41598-018-29059-5

**Published:** 2018-07-17

**Authors:** Saeedesadat Badieyan, Arezou Dilmaghani-Marand, Mohammad Javad Hajipour, Ali Ameri, Mohammad Reza Razzaghi, Hashem Rafii-Tabar, Morteza Mahmoudi, Pezhman Sasanpour

**Affiliations:** 1 Department of Medical Physics and Biomedical Engineering, School of Medicine, Shahid Beheshti University of Medical Sciences, Tehran, Iran; 2 Non-Communicable Diseases Research Center, Endocrinology and Metabolism Population Sciences Institute, Tehran University of Medical Sciences, Tehran, Iran; 3 Persian Gulf Marine Biotechnology Research Center, The Persian Gulf Biomedical Sciences Research Institute, Bushehr University of Medical Sciences, Bushehr, Iran; 4 Department of Urology, Shohada-e-Tajrish Hospital, Shahid Beheshti University of Medical Sciences, Tehran, Iran; 5 Department of Anesthesiology, Brigham and Women’s Hospital, Harvard Medical School, Boston, Massachusetts, 02115 United States; 6 School of Nanoscience, Institute for Research in Fundamental Sciences (IPM), Tehran, Iran

## Abstract

The polarization imaging technique is a powerful approach to probe microstructural and optical information of biological structures (*e.g*., tissue samples). Here, we have studied the polarization properties of different bacterial colonies in order to evaluate the possibility of bacterial detection and discrimination. In this regard, we have taken the backscattering Mueller matrix images of four different bacteria colonies (*i.e., Escherichia coli*, *Lactobacillus rhamnosus*, *Rhodococcus erythropolis*, and *Staphylococcus aureus*). Although the images have the potential to distinguish qualitatively different bacterial colonies, we explored more accurate and quantitative parameters criteria for discrimination of bacterial samples; more specifically, we have exploited the Mueller matrix polar decomposition (MMPD),frequency distribution histogram (FDH), and central moment analysis method. The outcomes demonstrated a superior capacity of Mueller matrix imaging, MMPD, and FDH in bacterial colonies identification and discrimination. This approach might pave the way for a reliable, efficient, and cheap way of identification of infectious diseases.

## Introduction

Identification, classification, and characterization of different species of bacteria are of great importance in microbiology and medicine and, therefore, extensive research has been conducted on development of new approaches for bacterial detection and discrimination^1–7^.

Considering their non-destructive nature, speed and much lower price, the optical based techniques have been at the center of scientific attraction. In this regard, surface plasmon resonance, fluorescent based methods and light scattering techniques are the most well-known optical methods^8–14^. The polarization based imaging techniques have received considerable attention due to their viability in characterization and analysis of a variety of material, textile, biomedical samples, and tissues^15–18^. The fundamental advantages of this technique including non-invasiveness, and real-time characterization have made the polarization imaging a promising technique in a wide range of medical applications^15^. Polarization images contain valuable abundant structural and optical information of the sample which cannot be obtained directly from intensity or spectral images^19–22^.

Although genetic polymorphism and biochemical characteristics of bacteria are enough to determine their evolutionary relationship, their morphological features can also be used for a rapid and cheap identification of these organisms^23^. It is well-recognized that bacterial morphologies are not random but have a reproducible biological relevance. Bacteria display extensive diveristies in terms of their shapes and arrangements. Based on their basic shapes, they are classified into three categories: *coccus*, *bacillus* and *spiral*^24,25^.

Bacterial-specific arrangement is a key characteristic that can be used for bacterial characterization. Bacteria arrange themselves side by side depending on the way they divide. For example, bacteria remain in pair, chain, groups of four, groups of eight or cluster after division. Based on their arrengement characteristics, bacteria form different colony patterns on Luria Broth agar media^23^. Therefore, it is possible to identify and descriminate between different bacterial species based on their morphology, arrangement and consequent colony patterns.

As bacterial colony pattern is still an unusual concept, its importance has not yet been discovered in different fields of medicine. The morphology of bacterial colony pattern is strongly dependent on the bacterial species and intercellular communication^26–28^. In some cases, the bacterial colony pattern is used as an excellent experimental characteristic to study multicellular interaction. A deep underestanding of the bacterial colony pattern has a critical capacity to shed more light on the important questions of genetics and morphogenesis^26,29^.

Recently, as a comprehensive description of polarization property, the Mueller matrix polarimetry was applied for characterization of various types of tissues^30–33^. Based on the polarimetric imaging technique, we have exploited its potential capacity to study the polarization properties of different bacterial colonies. Our study is based on obtaining the Mueller matrix with different polarization states of four different types of bacteria colonies including *Escherichia coli*, *Lactobacillus rhamnosus*, *Rhodococcus erythropolis*, and *Staphylococcus aureus*.

Considering anisotropic samples, the Mueller matrix elements are sensitive to the orientation of the samples which makes the quantitative characterization difficult and time consuming^34,35^. Two quantitative methods have been introduced to quantify the polarization properties, which have the advantages of orientation insensitivity and providing quantitative criteria (especially for biomedical diagnosis) that reveal the morphology and structure of samples. The first method is based on the polar decomposition of the Mueller matrix^36^. In this approach, the post processed Mueller matrix images will be analyzed and performing the required analysis, various polarization parameters of each species is derived.

The second method is based on the statistical analysis for obtaining the frequency distribution histograms (FDHs) of Mueller matrix images and their central moments^37^.

To the best of our knowledge there has been no study on the polarization properties of bacterial colonies accordingly. The results of our study demonstrated that the polarization properties and central moments’ values of different bacterial colonies are distinct. The difference in the polarization properties and central moments’ values of each colony is mainly originated from the distinct morphology and structure of each colony. Different polarization properties of various colonies have the potential to be used for the detection and classification of different species accordingly.

## Results and Discussion

**Figure 1:**
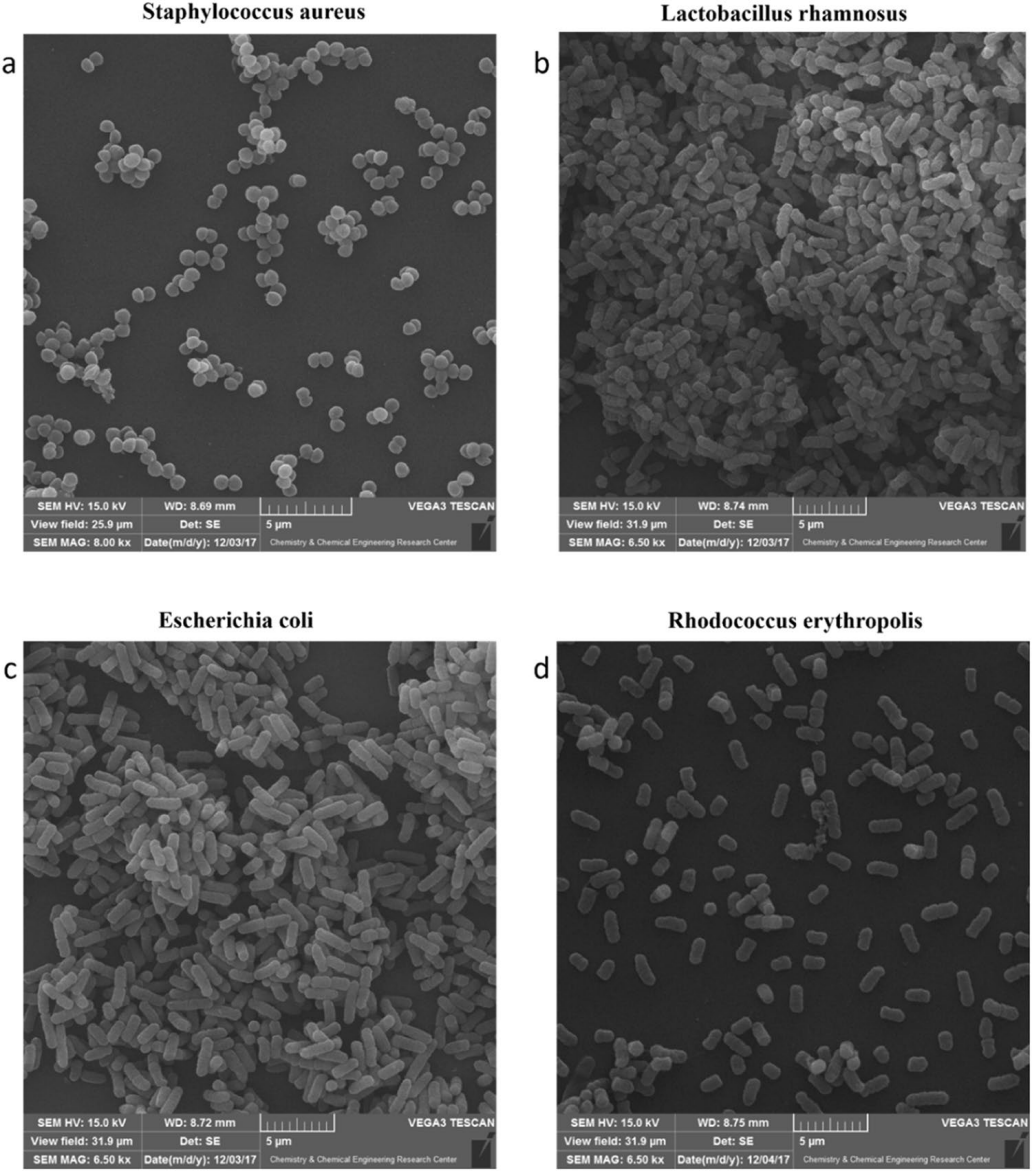
SEM images of morphology, arrangement and colony patterns of *Staphylococcus aureus*, *Lactobacillus rhamnosus*, *Escherichia coli* and *Rhodococcus erythropolis*.

Initially, for evaluating the performance of the system, the calibration test by measuring the scattering/backscattering Mueller matrix for the known samples (*e.g*., air and mirror) was performed. In order to calibrate the system, a linear polarizer and air was used as the sample. The error of the system was considered as the maximum difference in each component of Mueller matrix (measured) with the expected values for air and linear polarizer.The results of the calibration tests demonstrated that the errors of all Mueller matrix elements were less than 3%. The morphology, arrangement and colony pattern of *Escherichia coli*, *Lactobacillus rhamnosus*, *Staphylococcus aureus* and *Rhodococcus erythropolis* bacteria were evaluated using scanning electron microscopy (SEM) imaging. As shown in Fig. 1a, cocci-shaped *Staphylococcus aureus* are arranged in a particular pattern. Different colony patterns of the rod-shaped *Lactobacillus rhamnosus*, *Escherichia coli* and *Rhodococcus erythropolis* were detected in Fig. 1b–d respectively.

**Figure 2:**
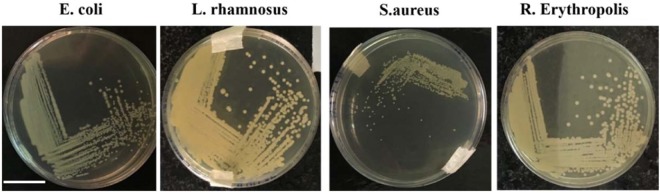
Colony patterns of *Escherichia coli*, *Lactobacillus rhamnosus*, *Staphylococcus aureus* and *Rhodococcus erythropolis* grown on the LB agar medium. Scale bar is 20 mm.

These bacteria also showed different colony patterns in LB agar medium. Figures 2a–d, respectively, show the colony patterns of *E. coli*, *L. rhamnus*, *S. aureus* and *R. erythropolis* grown on the LB agar medium. These colonies grow at same condition and obtained after 24 hours.

**Figure 3:**
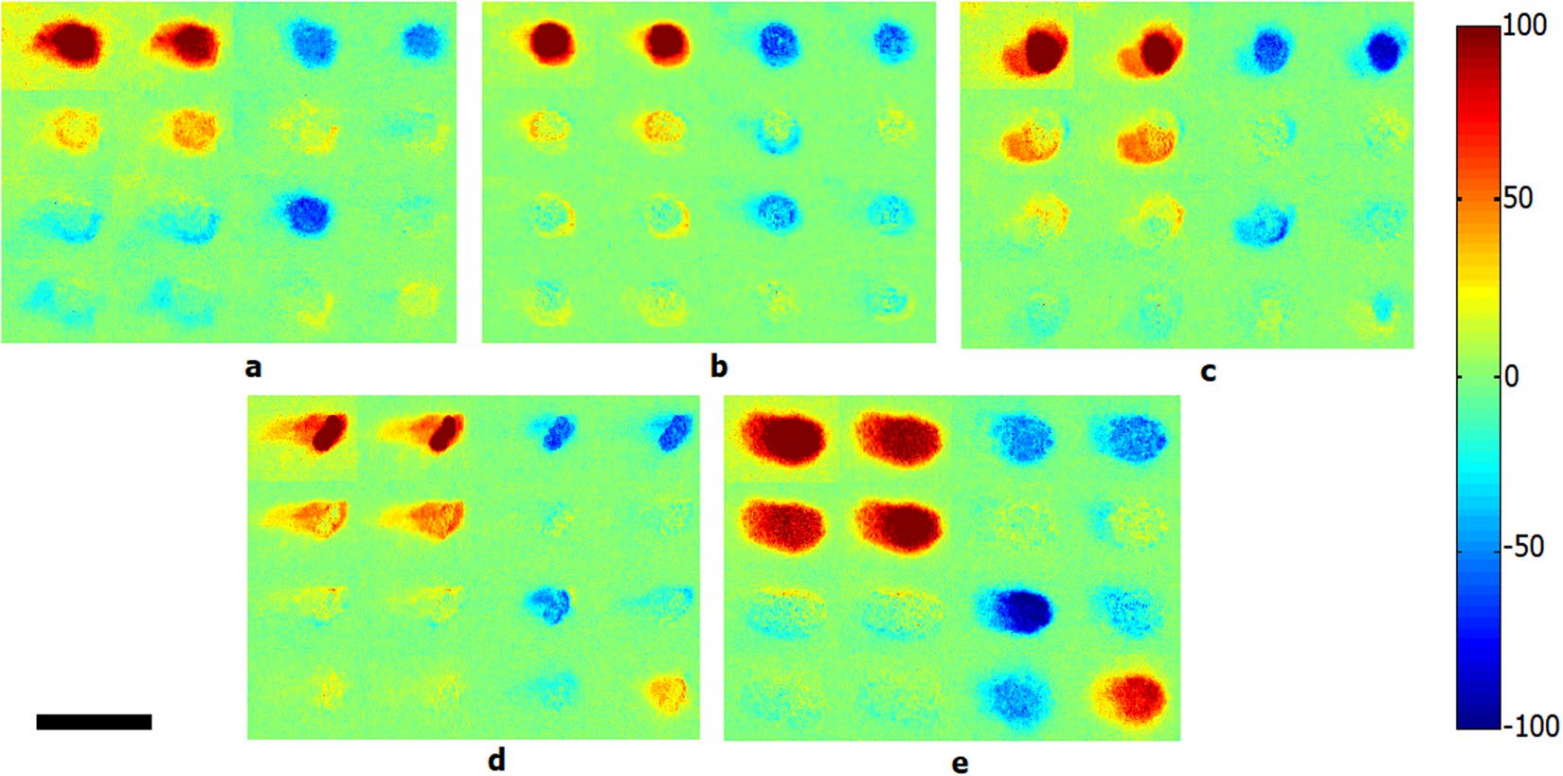
Calculated backscattering Mueller matrix images of four different bacterial colonies, (**a**) *Escherichia coli*, (**b**) *Lactobacillus rhamnosus*, (**c**) *Rhodococcus erythropolis*, (**d**) *Staphylococcus aureus* and (**e**) BFLBAM. Scale bar is 2 mm.

**Figure 4:**
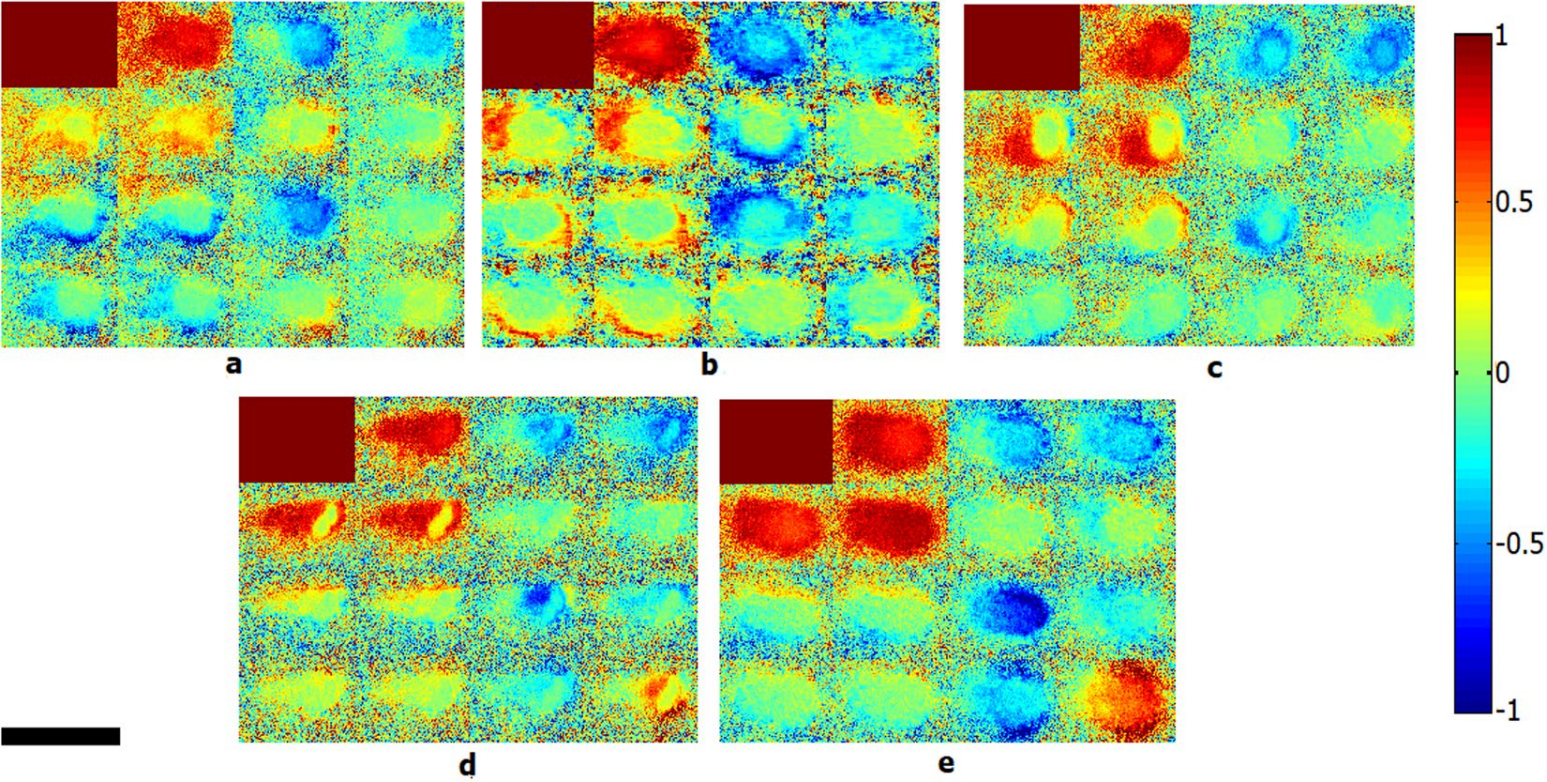
Calculated backscattering normalized Mueller matrix images of four different bacterial colonies, (**a**) *Escherichia coli*, (**b**) *Lactobacillus rhamnosus*; (**c**) *Rhodococcus erythropolis*; (**d**) *Staphylococcus aureus* and (**e**) LB agar medium. Scale bar is 2 mm.

Figures 3 and 4 show the images and normalized images of experiments for the calculated backscattering Mueller matrix of four different kinds of bacterial colonies (grown on the LB agar medium) and bacteria-free LB agar media (BFLBAM). In the normalized Mueller matrix, all elements are divided by m_00_ matrix accordingly.

Considering images of Figs 3 and 4 regarding the Mueller matrix elements of bacterial colonies and the BFLBAM, the characteristic behavior of the Mueller matrix elements provides primitive information for differentiation of species. Generally, for a sample with a low depolarization power, the values of the diagonal elements (m22, m33 and m44) will be larger than the other elements^38^. Based on the results of Figs 3 and 4, the BFLBAM reveal the largest diagonal elements, resembling the smallest depolarization power. This result can be associated with the size of the scattering particles (smaller than wavelength) in the BFLBAM, comparing with the size of the scattering centers in colonies (the bacteria with the size of several order of wavelength). Regarding bacterial colonies, the degree of depolarization can be determined from the Mueller matrix images accordingly. By the way, as the values of the depolarization are close, the qualitative analysis will not be adequate. In addition, by exploiting the information of the Mueller matrix, the isotropic properties of the sample can be evaluated accordingly. For isotropic samples, the Mueller matrices have only diagonal values, and the values of the m22 and m33 elements are equal to each other^38^. For anisotropic samples, the diagonal values are not equal and also there are non-diagonal elements in the Mueller matrices^38^. In this regard, the anisotropic nature of the samples are clearly confirmed, while the quantitative and accurate comparison is not possible. For a more accurate and quantitative analysis the Mueller matrix polar decomposition (MMPD) and central moments analysis methods are used to provide more explicit parameters for the optical properties of samples.

**Figure 5:**
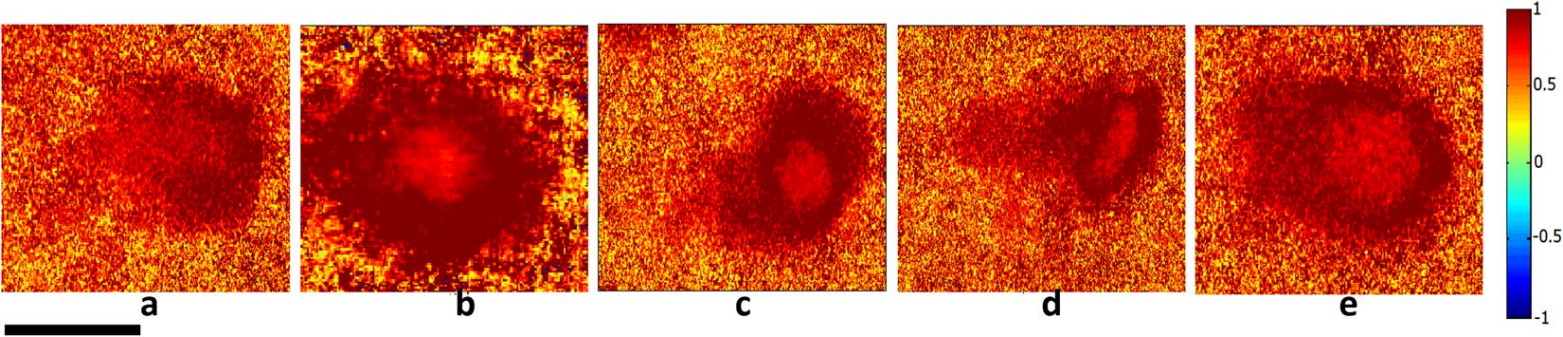
Two-dimensional intensity distributions of diattenuation for (**a**) *Escherichia coli*, (**b**) *Lactobacillus rhamnosus*; (**c**) *Rhodococcus erythropolis*; (**d**) *Staphylococcus aureus*; (**e**) BFLBAM. Scale bar is 1 mm.

**Figure 6:**
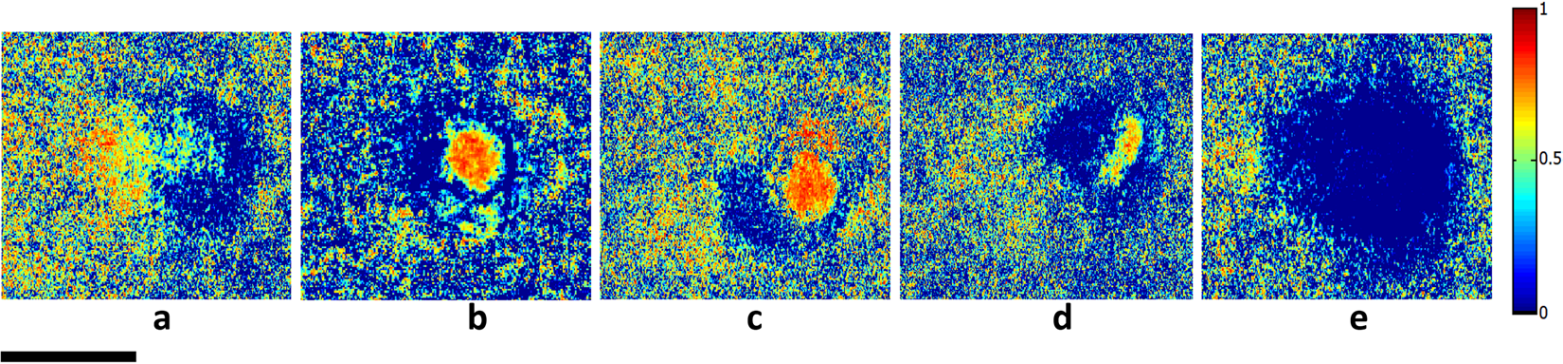
Two dimensional intensity distribution of depolarization for (**a**) *Escherichia coli*, (**b**) *Lactobacillus rhamnosus*; (**c**) *Rhodococcus erythropolis*; (**d**) *Staphylococcus aureus*; (**e**) BFLBAM. Scale bar is 1 mm.

**Figure 7:**
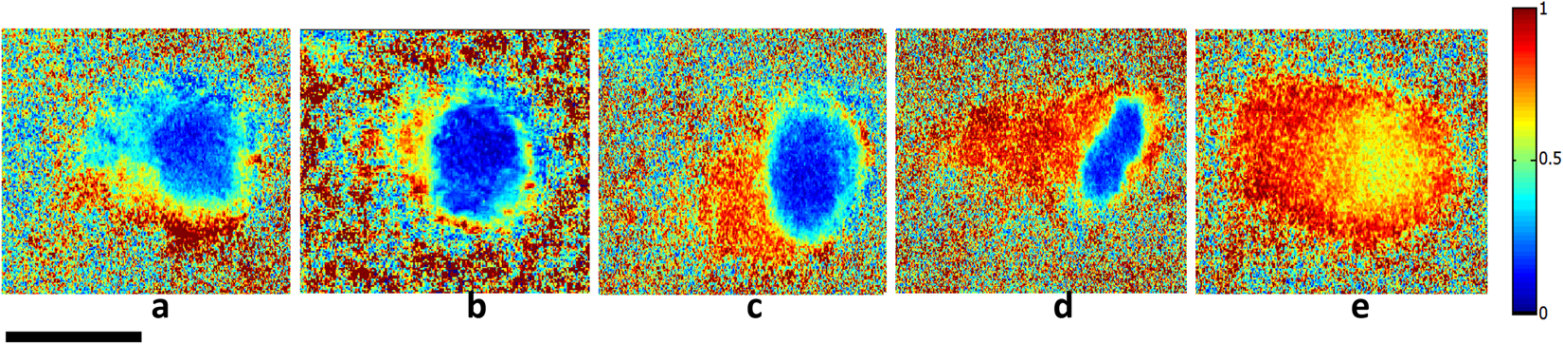
Two dimensional intensity distribution of polarizance for (**a**) *Escherichia coli*, (**b**) *Lactobacillus rhamnosus*; (**c**) *Rhodococcus erythropolis*; (**d**) *Staphylococcus aureus*; (**e**) BFLBAM. Scale bar is 1 mm.

Using the Lu–Chipman algorithm for polar decomposition of the Mueller matrix M, polarization images including diattenuation *D*, depolarization power Δ, and polarizance P was calculated. The images of M, D, Δ ans P have been obtained by processing each pixel of images based on the algorithm. Figures 5–7 illustrate two-dimensional intensity distribution of diattenuation, depolarization and polarizance images for four different bacterial colonies and BFLBAM accordingly.

In order to check the repeatability of the results, the set of measurements for every sample was repeated multiple times. Table 1 shows the mean value of each polarization parameter of each bacterium and BFLBAM accordingly.

**Table 1:** Mean values of the element of Mueller matrices obtained for four different Bacteria and BFLBAM.

Polarization parameter	Samples				
	*Escherichia coli*	*Lactobacillus rhamnosus*	*Rhodococcus erythropolis*	*Staphylococcus aureus*	BFLBAM
Diattenuation	0.8591	0.8847	0.9055	0.8451	0.8232
Depolarization	0.4506	0.6640	0.5580	0.5880	0.0372
Polarizance	0.1756	0.1245	0.1623	0.1527	0.6305

**Figure 8:**
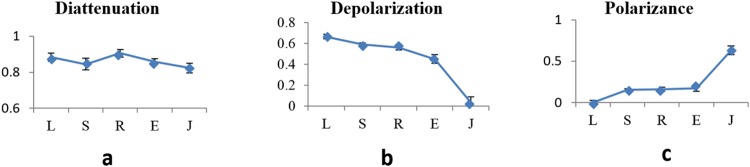
Comparison between the results of these five samples. (**a**) Diattenuation, (**b**) depolarization, and (**c**), polarizance parameters for five samples with errorbars, *S, L, R, E and J represent the *Escherichia coli*, *Lactobacillus rhamnosus, Rhodococcus erythropolis*, *Staphylococcus aureus*, and BFLBAM (respectively).

The results of Table 1 considering five samples are shown in Fig. 8. Figures 8a,b,c, demonstrate the diattenuation, depolarization, and polarizance for five samples. The error bars have been calculated based on the standard deviation of multiple measurements.

The MMPD method decomposes the Mueller matrix and drives the corresponding polarization optical parameters such as diattenuation, depolarization and polarizance. Comparison with the images of Figs 5–7, Table 1 and Fig. 8 show more precise comparison between polarization properties of samples. It can be observed from Figs 5–7 and Table 1 that BFLBAM has the lowest diattenuation and depolarization and the highest polarizance. This result is due to the significant structural difference between BFLBAM and bacterial colonies. Since the BFLBAM is clearer than bacterial colonies, the diattenuation due to the absorption of light in the BFLBAM will be less than in bacterial colonies. Furthermore the structure of BFLBAM will cause lesser depolarization comparing with bacterial colonies. Figures 8b,c show the reverse trend of depolarization and polarizance accordingly. Considering the microscopic structures, the results show that the density and shape of bacterial colony structures are the main reason for differences in their polarization properties. It is well recognized that bacteria have density fluctuations in their colony patterns^39^. The bacteria colony size, on the agar plate, is strongly dependent on their density. Chapuis *et al*.^40^, demonstrated that bacteria with low density have large colony size while those having large density are usually small. In addition, the growth rate of bacteria determines their colony size and density. Due to their different growth rate, *E. coli* and *R. erythropolis* have different density and colony size on the agar plate. For example, *E. coli* bacterium having higher growth rate showed larger density and smaller colony size compared to *R. erythropolis*. In this regard and considering the results from SEM images in Fig. 1, the density of colony and shape of bacteria would influence the polarization properties of the colony; where in *E. coli* with higher density, the amount of depolarization is lower than R. erythroplis. This can be related to the fact that in lower densities, the individual scattering centers (bacteria) act more efficiently while in higher densities, the accumulation will somehow suppress the scattering. By the way this deduction might not be true in all cases which implies that there are other distinct polarization properties for each individual species.

**Figure 9:**
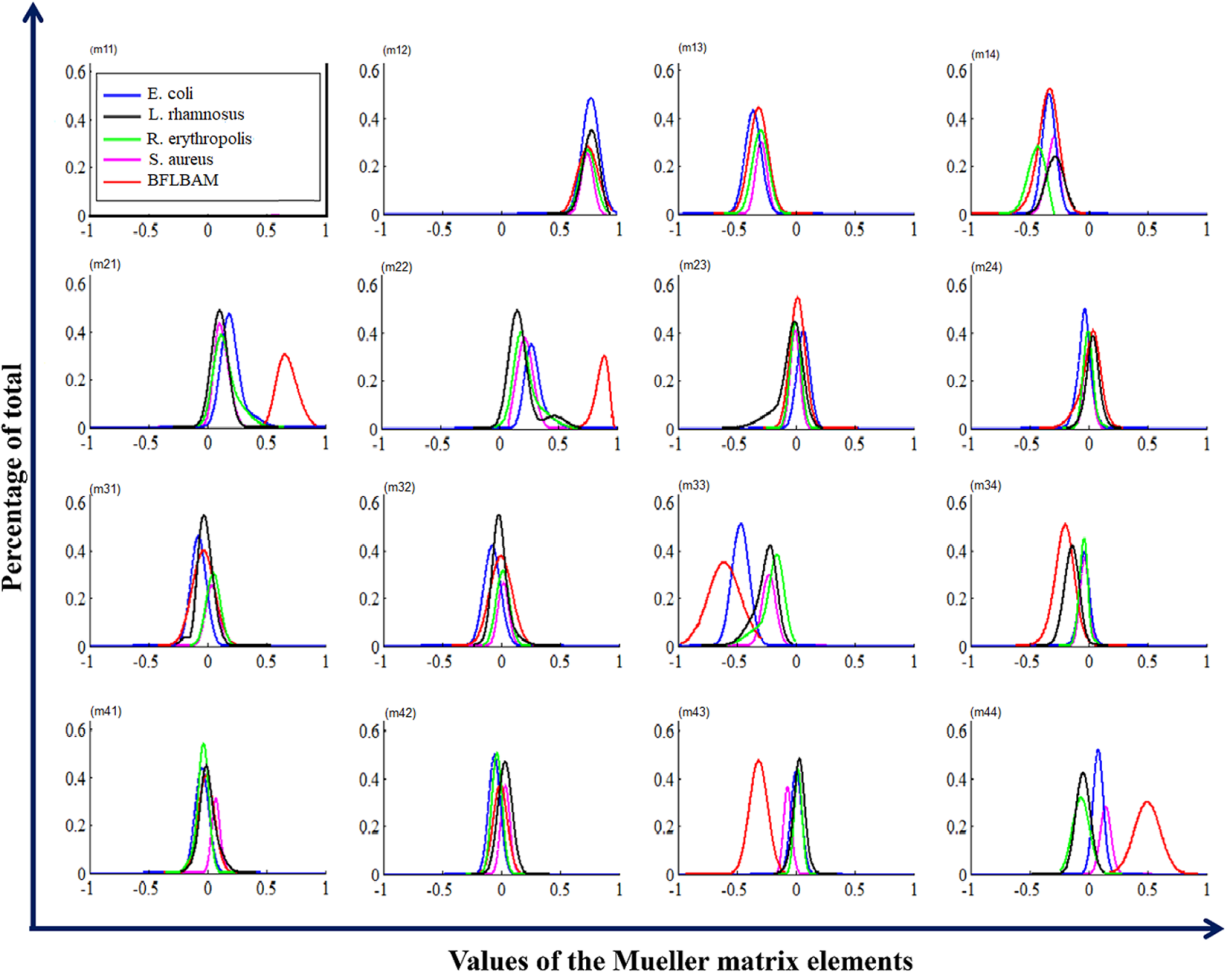
Frequency distribution histogram (FDH) of the Mueller matrix elements of different Bacteria samples and BFLBAM: *Escherichia coli* (blue line), *Lactobacillus rhamnosus* (black line), *Rhodococcus erythropolis* (green line), *Staphylococcus aureus* (magenta line), and BFLBAM (red line). The areas under the curves are normalized to 1.

Figure 9 represents the experimental results of the frequency distribution histogram (FDH) of the Mueller matrix elements; *Escherichia coli* (blue line), *Lactobacillus rhamnosus* (black line), *Rhodococcus erythropolis* (green line), *Staphylococcus aureus* (magenta line), and BFLBAM (red line). During the measurements, the angle between PSG and PSA arm are kept constant at the 30 deg.

FDHs of different samples have different distributions. Table 2 shows corresponding central moment parameters (mean and variance).

**Table 2:** Central moment parameters of the Mueller matrix elements for different samples.

	m12	m13	m14	m21	m22	m23	m24	m31	m32	m33	m34	m41	m42	m43	m44
S/P1	0.7322	−0.3	−0.309	0.1307	0.2305	−0.002	−0.004	0.0399	0.0242	−0.237	−0.034	0.0753	0.0393	−0.071	0.1572
L/P1	0.7614	−0.387	−0.291	0.1457	0.1948	−0.0501	0.0381	−0.012	−0.006	−0.262	−0.145	0.0057	0.0411	0.0318	−0.055
E/P1	0.7609	−0.362	−0.327	0.2072	0.2979	0.0638	−0.032	−0.083	−0.081	−0.472	−0.035	−0.046	−0.056	0.0059	0.0845
R/P1	0.7416	−0.315	−0.446	0.1636	0.2249	0.0001	0.0001	0.0609	0.0229	−0.205	−0.043	−0.038	−0.037	0.0186	−0.043
J/P1	0.7509	−0.306	−0.341	0.6799	0.8696	0.0179	0.0176	−0.029	0.0017	−0.631	−0.204	−0.009	−0.006	−0.323	0.496
S/P2	0.0024	0.0029	0.0035	0.0056	0.0054	0.002	0.0018	0.0028	0.002	0.0042	0.0018	0.0015	0.0015	0.0016	0.0027
L/P2	0.0039	0.0101	0.0052	0.0169	0.0166	0.014	0.0035	0.0059	0.005	0.0113	0.0047	0.0053	0.0045	0.0032	0.0046
E/P2	0.0047	0.0062	0.0045	0.008	0.0064	0.0034	0.0033	0.0081	0.0071	0.0056	0.0023	0.0039	0.0033	0.0023	0.0019
R/P2	0.0039	0.0058	0.0048	0.0123	0.0116	0.0024	0.0022	0.0042	0.0034	0.0119	0.0018	0.0028	0.0023	0.0017	0.0049
J/P2	0.0072	0.0082	0.0082	0.0072	0.0032	0.004	0.008	0.0108	0.0099	0.0265	0.0069	0.0047	0.004	0.0062	0.0133

*S, L, R, E and J represent the, *Escherichia coli, Lactobacillus rhamnosus, Rhodococcus erythropolis, Staphylococcus aureus*, and BFLBAM, respectively.

Each component of the FDH Mueller matrix in Fig. 9 contains of five curves for *Escherichia coli* (blue line), *Lactobacillus rhamnosus* (black line), *Rhodococcus erythropolis* (green line), *Staphylococcus aureus* (magenta line), and BFLBAM (red line). All values are measured at the same angles (The angle between PSG and PSA is considered 30 deg). The central moments (P1, P2, P3, and P4) are calculated based on the FDH curves of Fig. 9 and are listed in Table 2. The utilization of FDHs and central moments for samples offers quantitative and clearer information than intensity images^37^.

Comparing different results in Fig. 9 and Table 2, it can be concluded that different samples have different statistical behaviors (FDHs).

For all the samples it is observed that the corresponding FDH curves for m22 and m33 are different which indicates the anisotropic nature of the samples (e.g. for *Escherichia coli*, P1 of the m22 and m33 are 0.2979 and -0.472, p2 are 0.0064 and 0.0056). The greater difference between m22 and m33 indicates more anisotropy. Table 2 shows that differences in P1 of m22 and m33 elements for BFLBAM are considerable (0.8696 and −0.631) respectively. It indicates that BFLBAM is more anisotropic comparing with other bacterial colony samples. This is because those fibers in BFLBAM sample are well aligned in a specific direction. Among the bacteria samples, *Rhodococcus erythropolis* have the slightest difference between m22 and m33 and are therefore more isotropic than the other colonies. This fact can be relevant to the location of the bacteria inside the colony which is in a range of direction without any specific order. The larger P1 for the diagonal values of the Mueller matrix indicates the smaller depolarization power^37^. The results of Fig. 6 and Table 1 regarding the depolarization of different samples are in good accordance with the results of Fig. 8. In addition, based on the fact that the large P2 represents the distance of the polarization values from the mean value, the large distribution width of the FDHs, is considered as an indicator of the complexity of the samples^37^. Table 2 shows that for most elements, P2 for BFLBAM has larger values, which indicates a more complex structure in bacterial samples. For bacterial samples P2 values are of the same order which show the same complexity. The smaller positive and negative ranges of FDH curves for m24, m42, m34, and m43 elements indicate birefringent structures^37^. Considering results for different type of bacteria samples and BFLBAM, the above values are small and almost in a same range which indicates that in all samples there are birefringent structures, while in some elements BFLBAM shows a slight difference. In order to determine a more accurate relationship between the extracted parameters, P3 (skewness) and P4 (kurtosis) (which have significant different values for some elements) further investigations are required.

Based on the results of FDH, it can be concluded that some indicators such as m21, m22, and m34 are brilliant candidates of formation and existence of bacterial colony on the surface. By the way, m33 and m44 have the potential for differentiation of different bacterial colonies accordingly.

## Conclusions

Based on a polarization imaging system, the polarization properties of five samples including BFLBAM and bacterial colonies of *Escherichia coli, Lactobacillus rhamnosus, Rhodococcus erythropolis*, and *Staphylococcus aureus*, have been studied. Using the backscattering Mueller matrices of samples, and using the Mueller matrix polar decomposition (MMPD) method, FDH and central moment analysis method, we have analyzed the polarization based properties of the samples accordingly. The preliminary results of our study show that the acquired parameters (P1,P2) of methods (Muller matrix images. distribution curves and central moment parameters) might have the potential to give quantitative criteria to distinguish between different bacterial colonies. In order to understand the more accurate relationship between the obtained quantitative parameters and bacterial colonies characterization, more extensive structural studies and modeling using various bacterial colony samples are required.

## Methods

### Bacterial Samples Preparation

All bacterial species were separately grown in Luria Broth (LB) consisted of 1% tryptone, 0.5% yeast extract and 0.5% sodium chloride. In the next step, bacteria were separately cultured in solid LB agar medium containing 1% tryptone, 0.5% yeast extract, 0.5% sodium chloride and 1.5% agar and incubated at 37 °C overnight. All steps were performed under sterile condition. Immediately after 24 h, the patterns of bacterial colonies were analyzed.

### SEM Imaging

All bacteria were separately cultured in LB media and incubated at 37 °C for several hours to prepare a mid-log culture of each bacterial species. All bacteria in mid-log growth were harvested by centrifugation (at 2000 g for 5 min) and washed twice with phosphate buffer solution (PBS). The harvested cells were fixed with 3% glutaraldehyde at 4 °C for 4 hour, and washed with PBS for 2 min. This washing step was repeated for three times. In the next step, bacteria were dehydrated using graded series of ethanol (10%, 30%, 50% and 70%) for 15 min. Then, they were dehydrated with 90–100% ethanol and HMDS drying. After removing excess liquid (supernatant), bacteria dried in a fume hood for 24 h. Finally they were mounted on a SEM stub, coated with a layer of gold and imaged using a Tescan Vega3 SEM.

### Polarimetric Imaging Setup and Data Treatment

**Figure 10:**
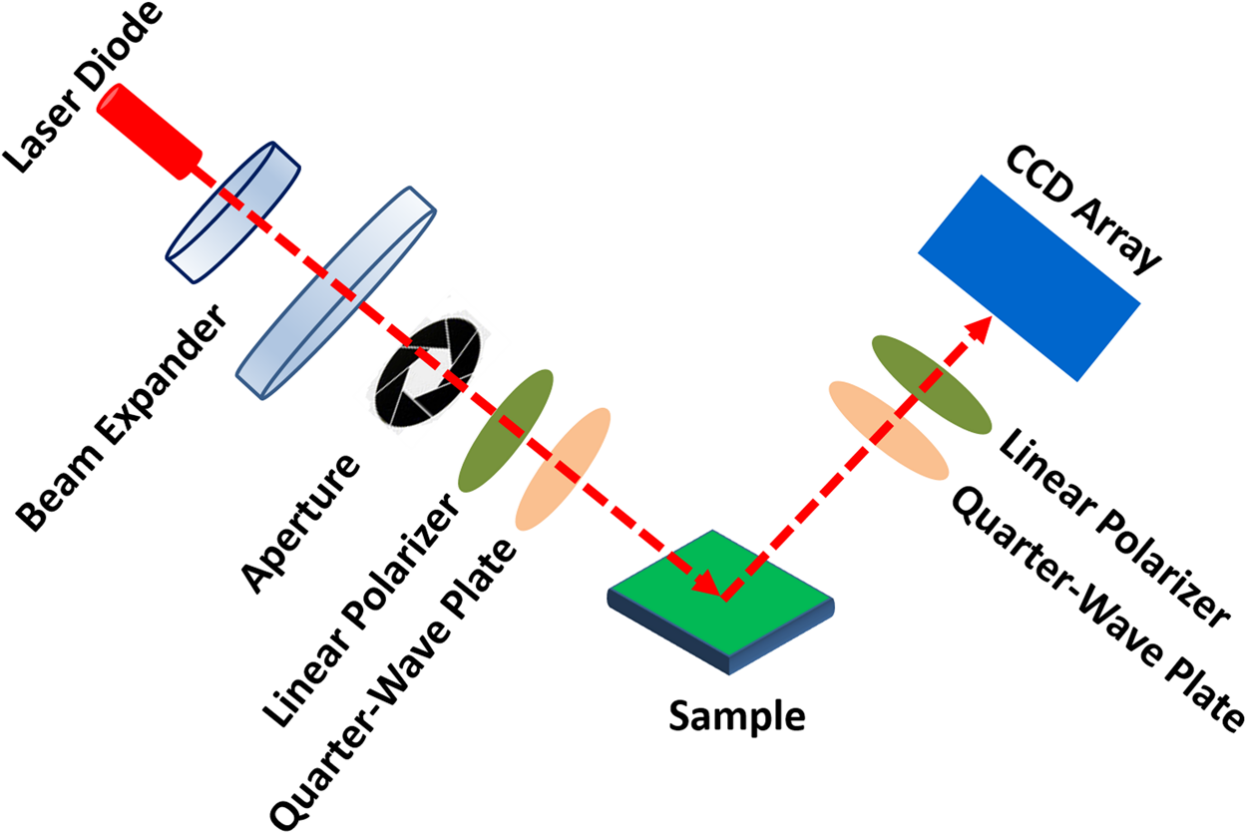
Schematic of the experimental setup for polarimetric imaging.

In order to acquire images for different polarization states, we established our set up as depicted schematically in Fig. 10. Generated coherent light from laser diode (5 mW, wavelength = 632.8 nm) was the source of illumination. In order to acquire the uniform illumination, the laser beam was passed through the beam expander and the diaphragm accordingly. The polarization state of illumination beam was determined by a Polarization State Generator (PSG) consisting of a linear polarizer and a quarter wave plate. The reflected light from the sample was passed through a Polarization State Analyzer (PSA) composed of the same element of PSG assembled in the reverse order. The angle between PSG and PSA arms was kept at 30° to avoid the surface reflection from the sample. A CCD detector collects the images from the sample in each case.

In order to obtain the Mueller matrix of samples, 36 intensity images (six different input (PSG) and output (PSA) polarization state) are required, as illustrated in Table 3. In Table 3, first and second indices denote input and output state of polarization. All the images are taken sequentially after changing the polarizer/analyzer configurations and the total procedure took 10 min. The recorded images were imported in MATLAB and raw images were processed. The 16 elemental Mueller matrix images are calculated based on the set of equation 1^41–43^. The system was analyzed for air and mirror and the resulted Mueller matrix was verified with 3% of error.

1 $$\begin{array}{c}M=[\begin{array}{cccc}{m}_{11} & {m}_{12} & {m}_{13} & {m}_{14}\\ {m}_{21} & {m}_{22} & {m}_{23} & {m}_{24}\\ {m}_{31} & {m}_{32} & {m}_{33} & {m}_{34}\\ {m}_{41} & {m}_{42} & {m}_{43} & {m}_{44}\end{array}]\\ =\,[\begin{array}{cccc}{I}_{HH}+{I}_{HV}+{I}_{VH}+{I}_{VV} & {I}_{HH}+{I}_{HV}-{I}_{VH}-{I}_{VV} & {I}_{PH}+{I}_{PV}-{I}_{MH}-{I}_{MV} & {I}_{RH}+{I}_{RV}-{I}_{LH}-{I}_{LV}\\ {I}_{HH}-{I}_{HV}+{I}_{VH}-{I}_{VV} & {I}_{HH}-{I}_{HV}-{I}_{VH}+{I}_{VV} & {I}_{PH}-{I}_{PV}-{I}_{MH}+{I}_{MV} & {I}_{RH}-{I}_{RV}-{I}_{LH}+{I}_{LV}\\ {I}_{HP}+{I}_{HM}-{I}_{VP}-{I}_{VM} & {I}_{HP}-{I}_{HM}-{I}_{VP}+{I}_{VM} & {I}_{PP}-{I}_{PM}-{I}_{MP}+{I}_{MM} & {I}_{RP}-{I}_{RM}-{I}_{LP}+{I}_{LM}\\ {I}_{HR}-{I}_{HL}\,+\,{I}_{VR}-{I}_{VL} & {I}_{HR}-{I}_{HL}-{I}_{VR}+{I}_{VL} & {I}_{PR}-{I}_{PL}-{I}_{MR}+{I}_{ML} & {I}_{RR}-{I}_{RL}\,-\,{I}_{LR}+{I}_{LL}\end{array}]\end{array}$$

**Table 3:** The complete 36 intensity measurement.

PSG path	PSA path					
	H	V	P	M	R	L
H	*I*_*HH*_	*I*_*HV*_	*I*_*HP*_	*I*_*HM*_	*I*_*HR*_	*I*_*HL*_
V	*I*_*VH*_	*I*_*VV*_	*I*_*VP*_	*I*_*VM*_	*I*_*VR*_	*I*_*VL*_
P	*I*_*PH*_	*I*_*PV*_	*I*_*PP*_	*I*_*PM*_	*I*_*PR*_	*I*_*PL*_
M	*I*_*MH*_	*I*_*MV*_	*I*_*MP*_	*I*_*MM*_	*I*_*MR*_	*I*_*ML*_
R	*I*_*RH*_	*I*_*RV*_	*I*_*RP*_	*I*_*RM*_	*I*_*RR*_	*I*_*RL*_
L	*I*_*LH*_	*I*_*LH*_	*I*_*LP*_	*I*_*LM*_	*I*_*LR*_	*I*_*LL*_

H: Horizontal polarization; V: Vertical polarization; P: +45° Linear polarization;

M: −45° Linear polarization; R: Right circular polarization; L: Left circular polarization.

### Analysis of Polarimetric Images

In order to summarize the polarization behavior of the samples, the optical parameters are derived from the analysis of images. The parameters are extracted according to the Lu–Chipman algorithm for polar decomposition of the Mueller matrix (M)^36^. As described by equation 2, the algorithm is based on the decomposition of M into three basis matrices for each pixel of image, namely, a depolarization (${M}_{{\rm{\Delta }}}$), a retardance (*M*_R_), and a diattenuation (*M*_D_):

2 $${\boldsymbol{M}}={{\boldsymbol{M}}}_{{\rm{\Delta }}}{{\boldsymbol{M}}}_{{\boldsymbol{R}}}{{\boldsymbol{M}}}_{{\boldsymbol{D}}}$$

The diattenuation (D) of the Mueller matrix is defined as equation 3

3 $$D=\frac{1}{{m}_{11}}\sqrt{{m}_{12}^{2}+{m}_{13}^{2}+{m}_{14}^{2}}$$

The polarizance of P is obtained by equation 4

4 $$P=\frac{1}{{m}_{11}}\sqrt{{m}_{21}^{2}+{m}_{31}^{2}+{m}_{41}^{2}}$$

The depolarization power (Δ) is given by equation 5

5 $${\rm{\Delta }}=1-\frac{|tr({M}_{{\rm{\Delta }}})-1|}{3}$$

where tr indicates the trace of the matrix.

### Central Moment Analysis of the Mueller Matrix Elements

In addition to the derived parameters of the Mueller matrix polar decomposition method, we have used statistical analysis method to obtain the frequency distribution histograms (FDHs) of the Mueller matrix images and their central moments to quantitatively measure the Mueller matrix elements^37,44^. Technically speaking, the FDHs show the distribution of intensity for each pixels of the image. In this regard each image of the Mueller matrix is converted to its FDH and the central moments of the FDH curves are calculated using equations 6–9^45^.

6 $$mean\,value=\mu =P1=E(X)$$

7 $$variance={\sigma }^{2}=P2=E[{(X-\mu )}^{2}]=Var(X)$$

8 $$skewness=\gamma =P3=\frac{E{(X-\mu )}^{3}}{{\sigma }^{3}}$$

9 $$kurtosis=Kurt[X]=P4=\frac{E{(X-\mu )}^{4}}{{\sigma }^{4}}$$

For a random variable $\,X$, P1 is the mean value of the FDH. P2 (variance) measures how far a set of random values are spread out from their mean value of the FDH. P3 (skewness) is a measure of the asymmetry of the FDH about its mean. The skewness value can be positive or negative. Negative (positive) skewness value indicates that the left (right) tail of the FDH is longer than right (left) tail. P4 (Kurtosis) considers the shape of the peaks of the FDH.

### Data availability

The datasets generated during and/or analysed during the current study are available from the corresponding author on reasonable request.
